# Dietary Protein and Amino Acid Profiles in Relation to Risk of Dysglycemia: Findings from a Prospective Population-Based Study

**DOI:** 10.3390/nu9090971

**Published:** 2017-09-04

**Authors:** Parvin Mirmiran, Zahra Bahadoran, Saeed Esfandyari, Fereidoun Azizi

**Affiliations:** 1 Department of Clinical Nutrition and Dietetics, Faculty of Nutrition Sciences and Food Technology, National Nutrition and Food Technology Research Institute, Shahid Beheshti University of Medical Sciences, P.O. Box 19395-4741 Tehran, Iran; mirmiran@endocrine.ac.ir; 2 Nutrition and Endocrine Research Center, Research Institute for Endocrine Sciences, Shahid Beheshti University of Medical Sciences, P.O. Box 19395-4763 Tehran, Iran; esfandyari29@gmail.com; 3 Endocrine Research Center, Research Institute for Endocrine Sciences, Shahid Beheshti University of Medical Sciences, P.O. Box 19395-4763 Tehran, Iran; azizi@endocrine.ac.ir

**Keywords:** amino acids, dietary protein, pre-diabetes, dysglycemia

## Abstract

Considering the limited knowledge on the effects of dietary amino acid intake on dysglycemia, we assessed the possible association of dietary protein and amino acid patterns with the risk of pre-diabetes in a prospective population-based study. Participants without diabetes and pre-diabetes (*n* = 1878) were recruited from the Tehran Lipid and Glucose Study and were followed for a mean of 5.8 years. Their dietary protein and amino acid intakes were assessed at baseline (2006–2008); demographic, lifestyle, and biochemical variables were evaluated at baseline and in follow-up examinations. Pre-diabetes was defined according to the American Diabetes Association criteria. Multivariate Cox proportional hazard regression models, adjusted for potential confounders, were used to estimate the risk of pre-diabetes across tertiles of dietary protein and amino acid pattern scores. The mean age of the participants (44.9% men) was 38.3 ± 12.7 years at baseline. Three major amino acid patterns were characterized: (1) higher loads of lysine, methionine, valine, aspartic acids, tyrosine, threonine, isoleucine, leucine, alanine, histidine, and serine; (2) higher loads of glycine, cysteine, arginine, and tryptophan; and (3) higher loads of proline and glutamic acid. Dietary total protein intake Hazard Ratio (HR) = 1.13, 95% Confidence Interval (CI) = 0.92–1.38 and HR = 1.00, 95% CI = 0.81–1.23, in the second and third tertile, respectively) was not related to the development of pre-diabetes. The highest score of second dietary amino acid pattern tended to be associated with a decreased risk of pre-diabetes (HR = 0.81, 95% CI = 0.65–1.01), whereas the third pattern was related to an increased risk in the fully adjusted model (HR = 1.24, 95% CI = 1.02–1.52; *p* for trend = 0.05). These novel data suggest that the amino acid composition of an individual’s diet may modify their risk of pre-diabetes.

## 1. Introduction

A pre-diabetes state, defined as impaired fasting glucose (IFG), impaired glucose tolerance (IGT), or impaired glycosylated haemoglobin, is closely related to development of type 2 diabetes and metabolic disorders [1]. The data suggests that dietary factors have an important role in the development of pre-diabetes and diabetes [2,3].

Currently, many misconceptions surround the role of dietary protein in the management of diabetes and pre-diabetes status [4,5]. Furthermore, limited and inconsistent data are available regarding the association of dietary protein intake and glucose/insulin homeostasis. Some beneficial short-term effects of dietary protein on insulin secretion in pancreatic β-cell function have been revealed in some investigations, whereas, in long-term population-based studies, an opposite direction was observed between protein intake and the risk of insulin resistance and type 2 diabetes [6,7]. Different protein sources have also shown different cardiometabolic outcomes [8,9,10].

The plasma profile of amino acids in relation to the risk of diabetes has been investigated in several previous studies [11,12,13]. However the possible role of dietary amino acid intakes in the development of dysglycemia and insulin resistance remains unknown. Nagata et al., in a longitudinal follow-up study, suggested that a high intake of branched chain amino acids (BCAAs) may be associated with a decrease in the risk of diabetes [14]. In contrast, Zheng et al., in the analysis of three cohort studies, reported that a high consumption of BCAAs was associated with an increased risk of type 2 diabetes [15].

In our previous study we indicated that amino acid patterns of diet were associated with the risk of cardiovascular disease (CVD), whereas total protein intake was not related to CVD events [16]. Considering the physiologically crucial role of amino acids in glucose/insulin metabolism and the current inconsistent recommendations regarding protein intake in the management of diabetes and pre-diabetes, we aimed to investigate the possible associations of amino acid patterns of diet and total protein intakes in relation to the risk of dysglycemia. We, therefore, conducted this prospective analysis in the framework of a national population-based study among a Middle Eastern population.

## 2. Methods 

### 2.1. Study Population

This study was conducted within the framework of the Tehran Lipid and Glucose Study (TLGS), an ongoing community-based prospective study being conducted to investigate and prevent non-communicable diseases, in a representative sample in the district 13 of Tehran, the capital city of Iran [17]. We recruited 3687 men and women, with complete dietary data, who participated in the third TLGS examination (2006–2008). Among the subjects aged 20 to 70 years (*n* = 2924), subjects with prevalent type 2 diabetes (T2D) at baseline (*n* = 321), missing data of anthropometrics, physical activity, or 2 h post challenge plasma glucose (*n* = 63) and participants who had under- or over-reports of total energy intakes (<800 kcal/day or >4200 kcal/day) (*n* = 284), were excluded. The participants who had no follow-up after the baseline examination (*n* = 117) were also excluded. The participants who had impaired fasting glucose (IFG) (fasting plasma glucose (FPG) levels 100 mg/dL to 125 mg/dL) or impaired glucose tolerance (IGT) (two hour values in the oral glucose tolerance test (OGTT) of 140 mg/dL to 199 mg/dL) were considered as pre-diabetic subjects [1]. Type 2 diabetes (T2D) was defined as FPG ≥ 126 mg/dL or 2-h post challenging plasma glucose (2h-PCPG) ≥ 200 mg/dL or as taking anti-diabetic medications. After the exclusion of pre-diabetes patients at baseline (*n* = 236) and participants with developed diabetes without preceding pre-diabetes (*n* = 25), the remaining participants, including 1878 adult men and women, were followed up to the fourth (2009–2011) and fifth TLGS examinations (2012–2014), ~3 years apart, for a median of 5.8 years from the baseline examination.

### 2.2. Ethical Consideration

Written informed consent was obtained from all participants. The study protocol, based on the ethical guidelines of the 1975 Declaration of Helsinki, was approved by the Ethics Research Council of the Research Institute for Endocrine Sciences, Shahid Beheshti University of Medical Sciences (Code: IR.SBMU.ENDOCRINE.REC. 1395.330).

### 2.3. Demographic and Anthropometric Measures

The demographic, anthropometric, and biochemical data were assessed at baseline (2006–2008). Trained interviewers collected information, including demographic data, medical history, medication use, and smoking habits, using pretested questionnaires. Weight was measured to the nearest 100 g using digital scales, while the subjects were minimally clothed, without shoes. Height was measured to the nearest 0.5 cm, in a standing position without shoes, using a tape meter. Body mass index was calculated as weight (kg) divided by the square of the height (m^2^). Waist circumference was measured to the nearest 0.1 cm, midway between the lower border of the ribs and the iliac crest at the widest portion, over light clothing, using a soft measuring tape, without any pressure to the body. For measurements of both systolic (SBP) and diastolic blood pressure (DBP), after a 15-min rest in the sitting position, two measurements of blood pressure were taken on the right arm using a standardized mercury sphygmomanometer; the mean of the two measurements was considered to be the blood pressure of the participant.

### 2.4. Biochemical Measures

Fasting blood samples were collected after 12 to 14 h from all study participants, at both baseline and the follow-up examination. Serum creatinine levels were assayed using the kinetic colorimetric Jaffe method. Fasting plasma glucose (FPG) was determined by the enzymatic colorimetric method using glucose oxidase. The standard two-hour plasma glucose (2-h PG) test was performed for all individuals who were not prescribed anti-diabetic drugs. Serum creatinine levels were assayed using the kinetic colorimetric Jaffe method. Triglyceride (TG) levels were assessed by enzymatic colorimetric analysis with glycerol phosphate oxidase. High-density lipoprotein cholesterol (HDL-C) was measured after the precipitation of apolipoprotein B, containing lipoproteins with phosphotungstic acid. The analyses were performed using Pars Azmoon kits (Pars Azmoon Inc., Tehran, Iran) and a Selectra 2 auto-analyzer (Vital Scientific, Spankeren, Netherlands). Both the inter-assay and intra-assay coefficients of variation of all assays were <5%.

### 2.5. Assessment of Amino Acid Intakes

A 168-item food frequency questionnaire (FFQ) was used at the first examination to assess an individual’s typical food intake over the previous year. The validity of the FFQ was previously assessed in a random sample by comparing the data from two FFQs, completed one year apart; reliability was assessed by comparing the data from the two FFQs and 12 patient self-report dietary recalls. The validity and reliability of the FFQ for dietary protein were acceptable; the correlation coefficients between the FFQ and multiple recalls were 0.65 and 0.50, and those between the two FFQs were 0.79 and 0.69 in male and female subjects, respectively [18]. A study of the reliability, comparative validity, and stability of dietary patterns derived from the FFQ also showed that there was reasonable reliability and validity of the dietary patterns among the population over time [19]. Trained dieticians asked participants to designate their intake frequency for each food item consumed during the past year on a daily, weekly, or monthly basis. The portion sizes of the consumed foods, reported in household measures, were then converted to grams.

The energy and nutrient contents of foods and beverages were analysed using the US Department of Agriculture Food Composition Table (FCT) because the Iranian FCT is incomplete and has limited data on the nutrient content of raw foods and beverages [20]. The intake of amino acids was also derived from the USDA FCT. Values for 18 individual amino acids were assigned to each of the FFQ food items, and amino acid intakes were calculated as the frequency of consumption of each food item multiplied by the amino acid content of the food.

### 2.6. Definition of Terms

The diabetes risk score (DRS) was calculated as follows: Systolic blood pressure (SBP) (mm Hg) <120 (0 points), 120 < SBP < 140 (three points), SBP ≥ 140 (seven points); family history of diabetes (five points); waist to height ratio (WHtR): <0.54 (0 points), 0.54–0.59 (six points), ≥0.59 (11 points); triglyceride to HDL-C ratio (TG/HDL-C): <3.5 (0 points), ≥3.5 (three points); fasting plasma glucose (FPG) (mmol/L): <5 (0 points), 50–5.5 (12 points), 5.6–6.9 (33 points) [21].

### 2.7. Statistical Methods

The mean (SD) values and the frequency (%) of the baseline characteristics of the study participants with and without the development of pre-diabetes were compared usingan independent *t*-test or a chi-square test, respectively.

To determine patterns of amino acid intakes, the principle component analysis (PCA) was conducted based on 18 amino acids (histidine, arginine, isoleucine, leucine, lysine, methionine, phenylalanine, threonine, tryptophan, valine alanine, aspartic acid, cysteine, glutamic acid, glycine, proline, serine, and tyrosine). Due to a high correlation between the amino acids, the promax rotation (an oblique rotation) was used. Considering eigenvalues >0.3, the scree plot, and the interpretability of the factors, three patterns were obtained. Amino acids with an absolute component loading ≥0.50 were selected to describe the pattern, although all amino acids contributed to the calculation of the pattern score. The Kaiser-Mayer-Olkin statistic, considered a measure of sampling adequacy, was 0.89, indicating a good appropriateness of factor analysis. To evaluate whether the correlation matrix was suitable for factor analysis, Bartlett’s test of sphericity was used; the *P* value for Bartlett’s test of sphericity was <0.001.

The factor scores of the participants were calculated using the sum of multiplying the intake of the standardized amino acids by their respective factor loadings on each amino acid pattern. The amino acid pattern scores were assessed as categorical (tertiles) variables in the models.

Cox proportional hazard regression models were used to assess the hazard ratios (HRs) and a 95% confidence interval (CI) of total dietary protein, as well as amino acid patterns, in relation to the risk of pre-diabetes. Two cox proportional hazard regression models were defined as follows: Model 1 was adjusted for age (year), sex (male/female), diabetes risk score (continuous), and physical activity (metabolic equivalent (MET)-h/week), and Model 2 was additionally adjusted for total energy intake (kcal/day) and dietary intake of protein (g/day). To assess the overall trends of HRs across tertiles of amino acids, the median of each tertile was used as a continuous variable in the Cox proportional hazard regression models.

All analyses were performed using SPSS for Windows version 20 (IBM Corp., Armonk, NY, USA) and STATA version 12 SE (Stata Corp LP, College Station, TX, USA), with a two-tailed *p* value < 0.05 being considered significant.

## 3. Results

The mean age of the participants (44.9% men) was 38.3 ± 12.7 years at baseline. During an average of 5.1 ± 2.1 years of follow-up, the incidence of pre-diabetes was 30.8%. The total protein intake was 87.1 ± 27.8 g/day, corresponding to a 13.7% to total energy intake. The top five contributing amino acids in the total protein intakes were glutamic acid (17.5%), aspartic acid (6.7%), leucine (6.5%), proline (6.4%), and lysine (5.0%), whereas tryptophan (0.88%) and cysteine (1.1%) made lower contributions. 

The baseline characteristics of the study participants, according to their status of pre-diabetes development, are presented in Table 1. Compared to the rest of cohort, participants with the development of pre-diabetes were more likely to be older and had higher BMI, WC, FPG, 2-PG, TG to HDL-C ratio, and blood pressure, as well as diabetes risk score at baseline (*p* for all < 0.05).

The dietary total protein intake (HR = 0.13, 95% CI = 0.92–1.38 and HR = 1.00, 95% CI = 0.81–1.23, in the second and third tertile, respectively) was not related to the development of pre-diabetes; moreover, no significant association was observed between animal- or plant-based protein intakes and the risk of pre-diabetes (HR = 0.86, 95% CI = 0.70–1.06 and HR = 1.18, 95% CI = 0.96–1.46, in the highest compared to the lowest tertile, respectively).

Principle component analysis identified three major amino acid patterns. Pattern 1 was characterized by higher loads of lysine, methionine, valine, aspartic acids, tyrosine, threonine, isoleucine, leucine, alanine, histidine, and serine; Pattern 2 had higher loads of glycine, cysteine, arginine, and tryptophan; and Pattern 3 had higher loads of proline and glutamic acid. These patterns explained 99.2% of the total variance in the amino acid intake overall (Table 2).

The risk (hazard ratios and 95% CI) of pre-diabetes development in relation to major amino acid patterns scores is shown in Table 3. The first pattern of dietary amino acid was not related to the incidence of pre-diabetes in the cox regression models, whereas the second pattern had a borderline negative association with the development of pre-diabetes, after the adjustment of all potential confounding variables (HR = 0.81, 95% CI = 0.65–1.01, in the second compared to the first tertile of the amino acid pattern score). The dietary amino acid pattern with a higher load of proline and glutamic acid was related to an increased risk of pre-diabetes in both the crude (HR = 1.30, 95% CI = 1.07–1.58; *p* for trend = 0.003) and fully adjusted models (HR = 1.24, 95% CI = 1.02–1.52; *p* for trend = 0.05).

## 4. Discussion

In this study, we observed that total protein intake was not related to the risk of dysglycemia in our population. However the remarkable result to emerge from our data was a significant direct association between an amino acid pattern with a higher load of glutamic acid and proline and the risk of pre-diabetes during six years of follow-up. A borderline inverse association was also observed between the amino acid pattern with a higher load of glycine, cysteine, arginine, and tryptophan and the risk of pre-diabetes. The second amino acid pattern had a significant correlation with dietary intakes of grains, meats, and legumes, whereas other patterns were highly correlated with dairy products; all the amino acid patterns had the same weak correlations with dietary intakes of fruits and vegetables.

It is noteworthy that studies investigating the association of dietary protein with insulin/glucose homeostasis, as well as the risk of dysglycemia and diabetes, have generated mixed results. Whereas some studies found that increased dietary protein intakes improve whole-body glucose metabolism, other studies found that higher intakes of protein either had no effect or might increase the risk of insulin resistance and diabetes. In a recent population-based study, a higher intake of total protein (OR = 2.13, 95% CI = 1.18, 3.81), as well as protein from animal (OR = 2.27, 95% CI = 1.18, 4.35) and red-meat (OR = 1.75, 95% CI = 1.14–2.68) sources, was accompanied with an increased risk of type 2 diabetes in women; these associations were mediated by insulin resistance [22]. A 10-year follow-up study also revealed that diabetes risk increased with higher total protein (HR = 2.15, 95% CI = 1.77, 2.60) and animal protein intake (HR = 2.18, 95% CI = 1.80–2.63) [6]. The reasons underlying the conflicting observations remain unclear. However sources of protein as well as the amino acid patterns of an individual’s diet may explain some of the inconsistencies. Recent studies showed that higher intakes of total and animal protein were associated with increased risks of diabetes, whereas higher plant protein intake tended to be associated with a lower risk of type 2 diabetes [9,10].

Few researchers have also addressed the possible role of dietary amino acid intake in the development of insulin resistance and dysglycemia, and previous works have only focused on the individual effect of dietary amino acids rather than their clustering effects. Zheng et al., in the meta-analysis of three cohort studies, including the Nurses’ Health Study (NHS; followed from 1980 to 2012), NHS II (followed from 1991 to 2011), and the Health Professionals Follow-up Study (HPFS; followed from 1986 to 2010), showed a higher risk of diabetes in relation to higher intakes of leucine, isoleucine, and valine; in this study, higher dietary BCAAs were significantly associated with higher plasma levels of these amino acids [15]. A recent study showed that BCAAs and aromatic amino acids, particularly tyrosine, may be novel aetiological mechanisms and contribute to an excess risk of diabetes among the Asian population [23]. In our study, the first amino acid pattern with a higher load of BCAAs was not related to the development of pre-diabetes.

Although the biologically plausible mechanisms for the adverse effects of high intakes of proline and glutamic acid on diabetes risk are less known, our findings regarding high-proline amino acid patterns support those of previous studies, which reported that high exposure to this amino acid may cause an impairment in insulin transcription and mitochondrial oxidative phosphorylation and consequently contribute to the β-cell dysfunction observed in type 2 diabetes [24]. Plasma levels of glutamate and proline were also shown to be strongly associated with hyperinsulinemia [25].

The prospective design of our study, as well as its relatively large sample size with long-term follow-up, detailed data on well-known diabetes risk factors and potential confounders, and comprehensive assessment of dietary intakes using a validated comprehensive FFQ, were the main strengths of the current investigations. Furthermore, use of DRS, composed of FPG, WHtR, TG to HDL-C ratio, family history of diabetes, and SBP, in multivariate models allowed us to take into account major diabetes risk factors without adding many variables that would lead to the instability of our models. The factor analysis-derived amino acid patterns used in our study provide us with a broader picture of the dietary amino acid composition in our population; this approach may also account for any inter-correlation among amino acids, consider their cumulative effects, and reduce their interdependent variations, compared to the individual amino acid approach. However, our study clearly had some limitations. Due to potential changes in an individual’s diet and changes in other risk factors of pre-diabetes during the study follow-up, some degree of misclassification might have occurred, which could lead to estimated hazard ratios biased towards null, as is inherent in any prospective study.

In conclusion, the evidence from this study suggests that a dietary amino acid pattern with a higher load of proline and glutamic acid may increase the risk of dysglycemia. A lack of significant relation between total dietary protein intake and the incidence of pre-diabetes in our study also points towards the idea that dietary amino acid composition may play a more important role than the amount of protein intake from a usual diet in relation to glucose metabolism.

## Figures and Tables

**Table 1:** Baseline characteristics of the participants.

Baseline Characteristics	Participants with Pre-diabetes (*n* = 578)	Participants without Pre-diabetes (*n* = 1299)	*p* Value
Age (year)	43.0 ± 12.1	36.3 ± 11.7	0.001
Male (%)	52.6	41.2	0.001
Body mass index (m^2^/kg)	27.8 ± 4.5	25.8 ± 4.5	0.001
Waist circumference (cm)	92.4 ± 11.3	85.5 ± 12.7	0.001
Systolic blood pressure (mm Hg)	112 ± 14.3	106 ± 13.1	0.001
Diastolic blood pressure (mm Hg)	74.4 ± 9.7	71.1 ± 9.9	0.001
Fasting blood glucose (mg/dL)	87.9 ± 6.2	83.7 ± 5.8	0.001
2h-blood glucose (mg/dL)	97.9 ± 20.5	87.5 ± 17.9	0.001
TG/HDL-C ratio	3.9 ± 2.6	3.0 ± 2.3	0.001
Family history of diabetes (%)	21.8	19.2	0.14
Diabetes risk score	12.7 ± 8.7	6.6 ± 7.2	0.001
Dietary protein (g/1000 kcal/day)	34.5 ± 6.0	34.2 ± 6.2	0.44

Data are mean ± SD unless stated otherwise; an independent t-test for continuous variables and a chi-square test for dichotomous variables were used.

**Table 2:** Component loadings for dietary amino acid patterns.

Amino acids	Patterns		
	1	2	3
Lysine	1.00	-	-
Methionine	0.77	-	-
Valine	0.70	-	-
Aspartic acid	0.73	-	-
Tyrosine	0.69	-	-
Threonine	0.68	-	-
Isoleucine	0.66	-	-
Leucine	0.65	-	-
Alanine	0.60	-	-
Histidine	0.58	-	-
Serine	0.51	-	-
Phenylalanine	-	-	-
Glycine	-	0.76	-
Cysteine	-	0.78	-
Arginine	-	0.72	-
Tryptophan	-	0.65	-
Proline	-	-	0.74
Glutamine	-	-	0.59
Variance (%)	94.7	2.6	1.8

Values are factor loadings of food patterns measured by factor analysis (*n* = 2369). Absolute values < 0.5 are excluded from the form for simplicity.

**Table 3:** The hazard ratio (95% confidence interval (CI)) of pre-diabetes across tertile categories of amino acid patterns scores

Amino Acid Pattern	T1	T2	T3	*p* for Trend
Pattern 1				
Crude	1.00	0.97 (0.79–1.20)	1.07 (0.87–1.31)	0.61
Model 1	1.00	0.97 (0.78–1.19)	1.06 (0.86–1.29)	0.67
Model 2	1.00	0.97 (0.85–1.30)	1.05 (0.78–1.19)	0.85
Pattern 2				
Crude	1.00	1.00 (0.82–1.22)	0.87 (0.71–1.07)	0.33
Model 1	1.00	0.92 (0.76–1.13)	0.83 (0.68–1.03)	0.24
Model 2	1.00	0.91 (0.74–1.11)	0.81 (0.65–1.01)	0.17
Pattern 3				
Crude	1.00	0.95 (0.77–1.17)	1.30 (1.07–1.58)	0.003
Model 1	1.00	1.00 (0.81–1.24)	1.24 (1.01–1.51)	0.04
Model 2	1.00	1.00 (0.81–1.24)	1.24 (1.02–1.52)	0.05

Amino acids were entered as g/day in the factor analysis. Cox regression models were used. Model 1: Adjusted for age, sex, physical activity, and diabetes risk score; Model 2: Additional adjustment for total energy intakes (kcal/day) and dietary intakes of protein (g/day).
